# Influence of Test Conditions on Sliding Wear Performance of High Velocity Air Fuel-Sprayed WC–CoCr Coatings

**DOI:** 10.3390/ma14113074

**Published:** 2021-06-04

**Authors:** Kaveh Torkashvand, Vinod Krishna Selpol, Mohit Gupta, Shrikant Joshi

**Affiliations:** Department of Engineering Science, University West, 46186 Trollhättan, Sweden; vinodselpol@gmail.com (V.K.S.); mohit.gupta@hv.se (M.G.); shrikant.joshi@hv.se (S.J.)

**Keywords:** tests conditions, sliding wear, HVAF, WC–CoCr, ball-on-disk, wear mechanism

## Abstract

Sliding wear performance of thermal spray WC-based coatings has been widely studied. However, there is no systematic investigation on the influence of test conditions on wear behaviour of these coatings. In order to have a good understanding of the effect of test parameters on sliding wear test performance of HVAF-sprayed WC–CoCr coatings, ball-on-disc tests were conducted under varying test conditions, including different angular velocities, loads and sliding distances. Under normal load of 20 N and sliding distance of 5 km (used as ‘reference’ conditions), it was shown that, despite changes in angular velocity (from 1333 rpm up to 2400 rpm), specific wear rate values experienced no major variation. No major change was observed in specific wear rate values even upon increasing the load from 20 N to 40 N and sliding distance from 5 km to 10 km, and no significant change was noted in the prevailing wear mechanism, either. Results suggest that no dramatic changes in applicable wear regime occur over the window of test parameters investigated. Consequently, the findings of this study inspire confidence in utilizing test conditions within the above range to rank different WC-based coatings.

## 1. Introduction

WC-based cermet coatings sprayed using high velocity thermal spray techniques are one of the most common protective layers employed in industry for wear applications [1,2]. They are expected to resist various wear environments, namely erosive wear, abrasive wear, fretting wear, fatigue and sliding wear [1,3]. There are several standardized test procedures to simulate different wear environments, including ASTM G76 [4] for erosive, ASTM G65 [5] for abrasive and ASTM G99 [6] for sliding wear conditions. Ball-on-disk is one of the most commonly employed tests to assess sliding wear performance of WC-based coatings [7,8]. In this test, however, there are several parameters that can potentially affect the results. It is known that a minor change in the contact conditions can influence the process of material removal in a tribosystem [9]. Bayer [10] remarks that “Wear is not a material property. It is a system response”, and this clearly highlights the importance of test conditions in a wear experiment. A sliding ball-on-disk test can result in different contact conditions depending on applied load, the material of the mating surfaces, hardness and size of the produced debris, which can potentially lead to changes in material removal mechanisms and, consequently, test results [11,12]. Occasionally, laboratory testing also compels conduct of multiple tests on a single specimen by varying the wear track radii [13,14]. For a given sliding distance, such tests inevitably lead to varying linear sliding velocities as well as different number of contact incidents at a specific location. The influence of the above on the test outcome also needs to be well understood. Further, it is pertinent to mention that the influence of test conditions on results could be material-specific. This is expected, since the threshold for a dramatic change in wear regime is expected to be intimately related to the materials being tested.

Performing ball-on-disc tests on thermally sprayed WC-based coatings typically results in a very shallow volume loss and, as a result, extremely low specific wear rate (in order of 10^−8^–10^−9^ mm^3^·N^−1^·m^−1^) [15,16]. This extremely low material removal introduces potential sensitivities to selection of test conditions as well as measurement procedure. As stated in the standard [6], chosen test parameters can influence precision of measurement as well as repeatability of test results. Particularly, great precision is required when volume loss is extremely low, as typically in the case of WC-based coatings. According to the standard ASTM G99, linear measures of wear scar should at least have a sensitivity of 2.5 µm. However, the depth of a typical wear track in a ball-on-disc test can be below 1 µm [17,18], which demands a higher measurement sensitivity than that stated in the standard. Generally, in a ball-on-disk test, applied load, test duration and sliding speed are the three main parameters that can potentially influence test results, particularly in terms of material loss and coefficient of friction. Some studies can be found in literature studying influence of these test parameters [19,20,21,22]. Based on the selected test parameters, two main regimes can be achieved during sliding wear test, namely mild wear and severe wear [1,23]. A transition is known to happen in wear regime from mild to severe by increasing load and/or sliding speed or running the test for longer distances. For instance, it has been shown in some studies [24,25] that a full transition in wear regime can occur in the case of HVOF-sprayed WC–Co samples when increasing the normal load from 19 to 35 N, resulting in a substantial increase in specific wear rate. Moreover, Wang et al. [26] performed ball-on-disk tests on high velocity oxy-fuel (HVOF)-sprayed WC–CoCr coatings at many different loads in the range 15 N–90 N for same test duration, sliding speed and ball material. They concluded that, despite a huge change in the applied load, the friction coefficient during the steady state period fluctuated in a small range of 0.3–0.4. However, their results showed that the mechanism of material removal can greatly change from carbide pull-outs to massive material exfoliation, depending on the applied load. Moreover, specific wear rate experienced a substantial increase by a factor of 10^2^ (from 3.6 × 10^−8^ to 3.5 × 10^−5^ mm^3^·N^−1^·m^−1^). Karaoglanli et al. [27] studied wear behaviour of HVOF WC–Co coatings, employing the ball-on-disk test under loads of 5 N and 15 N and sliding speeds of 10 and 20 cm/s for the same test duration and ball material. It was shown that an increase in sliding speed resulted in higher specific wear rate, while the wear rate decreased on increasing the normal load from 5 N to 15 N. Although these studies provide an understanding of the influence of test conditions on wear behaviour of WC–CoCr coatings, there is no published report systematically investigating the effect of all the aforementioned parameters on wear performance of WC-based coatings. In this study, we tried to fill this research gap by conducting a systematic study on wear behaviour of WC-based coatings under various wear conditions.

In order to have a good understanding of the role-playing factors and their effects on the sliding wear behaviour of WC-based coatings, conducting a comprehensive investigation is vital. This helps to establish a reliable testing routine for HVAF-sprayed WC–CoCr coatings. In this study, the effects of potential influencing parameters including angular velocity, load and sliding distance on wear behaviour of WC-based coatings, fabricated using high velocity air fuel (HVAF) spraying, are systematically evaluated and discussed.

## 2. Experimental Procedure

### 2.1. Deposition of Coating

Commercially available 86WC–10Co–4Cr powder manufactured employing agglomeration and sintering technique (trade name: Amperit^®^ 558.059, Höganäs GmbH, Goslar, Germany) was used as feedstock. Characteristics of the used powder are provided in Table 1. Domex 355 coupons of 25.4 mm diameter and 6 mm thickness were used as substrate. All the samples were degreased and mounted on a fixture rotating with a 1.66 m/s linear speed. The samples were grit blasted with alumina particles of average size 220 µm sprayed with the HVAF gun, resulting in a surface roughness (Ra) of approximately 4 µm. The grit-blasted substrates were then coated using a 5L2 convergent–divergent nozzle with an M3 HVAF torch (Uniquecoat Technologies LLC, Oilville, VA, USA). Spray parameters for grit-blasting and coating, both carried out using the above torch, are listed in Table 2.

### 2.2. Coating Characterization

Coated samples were ground and polished for microstructural characterization of coating cross-sections and hardness measurements. Surfaces of coated samples were also polished prior to wear testing. Following grinding using a 45 µm diamond disk, three steps of polishing were performed successively with 9 µm and then 3 µm Kemet liquid diamond media, followed by MasterMet 2 dispense to reach a mirror-polished state corresponding to *Ra* value less than 0.01 µm. General microstructure analysis of the coatings as well as post-wear analysis on wear scars were performed by scanning electron microscopy (SEM) (HITACHI TM3000 microscope, Krefeld, Germany, and ZEISS GeminiSEM 450, Oberkochen, Germany).

Vickers hardness measurement of the coating was conducted employing Struers Duramin-40 microhardness tester. Following the standard ASTM E384 [28], a total of fifteen indentations were performed on coating specimens.

### 2.3. Ball-on-Disk Sliding Tests

Samples were exposed to sliding wear employing a ball-on-disk testing rig (Tribometer TRB3, Anton-Paar, Buchs, Switzerland) following the procedure of the standard ASTM G99. The tests were conducted on mirror-polished samples. Three parameters, namely angular velocity, load and sliding distance, were varied (see Table 3). A test run conducted at 20 N of normal load for 5000 m of sliding distance with a linear speed of 0.2 m/s was selected as the reference. All the test runs were performed at a constant linear speed of 0.2 m/s, which resulted in various angular velocities corresponding to 2400, 2000, 1700, 1500 and 1333 rpm when the radii of the wear tracks, made by the sliding ball on the coating surface, were set to 5, 6, 7, 8 and 9 mm, respectively. That Alumina ball of radius 6 mm was used as the mating material in all cases.

**Figure 1:**
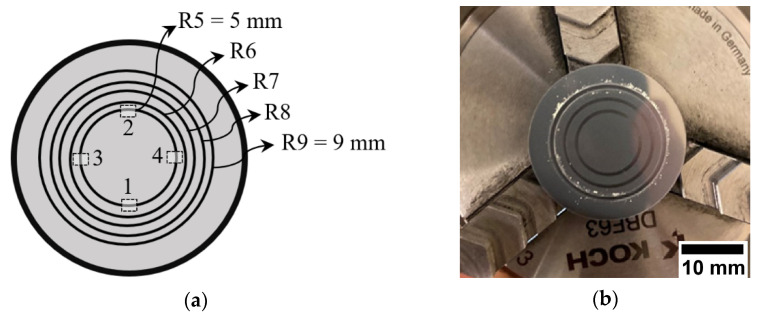
(**a**) Schematic diagram of different radii and distinct locations for volume loss measurements on the testing samples, and (**b**) a typical sample after ball-on-disk test.

For each set of tests to assess parametric impact on ensuing results, the parameter of interest was systematically varied compared to the reference run mentioned above. The angular velocity was changed by varying the wear track radius and keeping the linear speed constant during the tests. All the tests conducted to assess influence of angular velocity (Figure 1a) were repeated three times at each wear track radius on three different samples (S1, S2 and S3). Figure 1b shows a typical sample after testing on radii of 5, 7 and 9 mm. For the set of tests using the load as variable parameter, the normal applied load was varied between 20 N and 40 N. Due to limitation in the testing rig, it was not plausible to increase the load beyond 40 N. In yet another, the sliding distance was increased to 10 km from 5 km. Finally, in one particular run, both load and distance were simultaneously increased to 30 N and 10 km, respectively. The tests with load and distance as variables were conducted on radii of 7 and 8 mm. This was done since the outcome of the tests of different radii (see Section 3.2) has already established that the wear track radius does not influence the specific wear rate.

Sliding data such as friction force and friction coefficient were continuously monitored during the tests. After each test, the samples were ultrasonically cleaned and the volume loss measured employing white light interferometry (WLI) method (Profilm 3D, Filmetrics, Unterhaching, Germany). The measurement was performed at four different locations on the wear track, as shown in Figure 1a. The cross-sectional area of the wear track at each location was determined by dividing measured volume loss by length of wear track at each segment measurement. Approximate arc length of each segment is 1.75 mm. From the four measured values, an average value for the cross-section area of the whole wear track was calculated along with standard deviation of the four measurements. Having total length of the wear track and the average value of cross-section area, the total volume loss from the wear track (Equation (1)), and therefore, the specific wear rate, can be obtained according to Equation (2).

(1) $$V_{loss}=\frac{1}{2}\;\left(\pi .R\right).\left(\frac{V_{1}}{l_{1}}+\frac{V_{2}}{l_{2}}+\frac{V_{3}}{l_{3}}+\frac{V_{4}}{l_{4}}\right)$$

(2) $$W=\frac{V_{loss}}{L.d}\;$$

where in Equation (1) *V*_1_, *V*_2_, *V*_3_ and *V*_4_ are volume losses at the four locations shown in Figure 1a; *l*_1_, *l*_2_, *l*_3_ and *l*_4_ are the corresponding arc length of the segments; and *R* is radius of the wear track. *L* and *d* in Equation (2) are the applied normal load and total sliding distance, respectively.

## 3. Results and Discussion

### 3.1. Coatings Characterization

**Figure 2:**
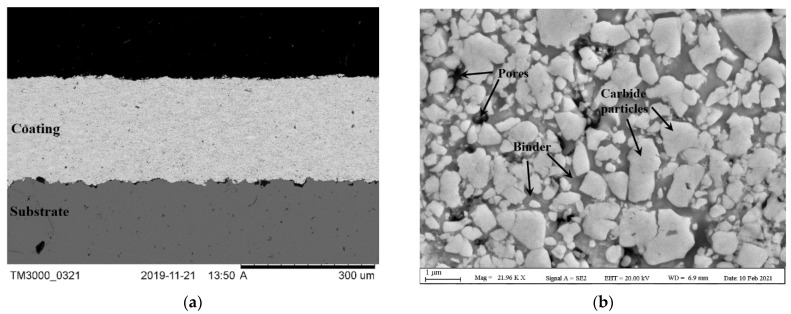
SEM images of cross-sections of deposited coatings at (**a**) low magnification and (**b**) high magnification.

Figure 2 shows low and high magnification images of the WC–CoCr coating used throughout this study. It can be seen that a uniform and dense WC–CoCr coating is achieved by the HVAF method. Although there are some submicron pores (as indicated in Figure 2) noted in the coating, the overall porosity content is less than 1 percent and comparable with literature [7,8]. Vickers hardness value for the coating measured to be 1410 ± 27 HV_0.3_. All the coating specimens utilized for subsequent ball-on-disk wear tests were simultaneously HVAF sprayed to ensure minimal variation, and the influence of each test parameter on ensuing results is individually discussed below.

### 3.2. Influence of Angular Velocity on Wear Behaviour

#### 3.2.1. Local Variations within a Wear Track

**Figure 3:**
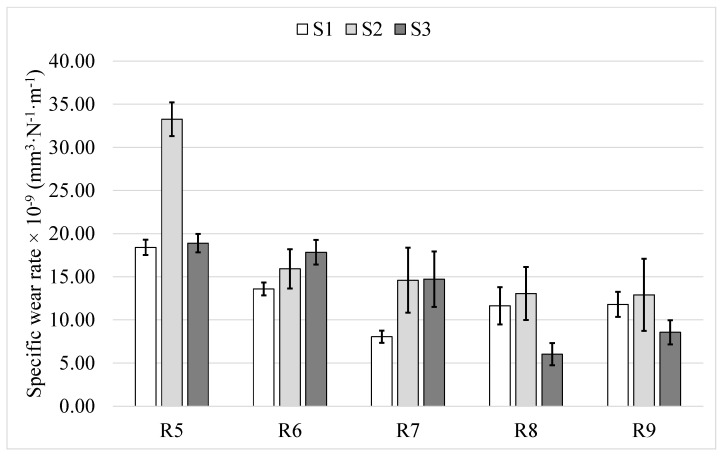
Specific wear rates from all fifteen ball-on-disc tests conducted at various wear track radii.

According to Table 3, identical coating specimens were subjected to ball-on-disk tests, with the track radius being the only variable. As mentioned in Section 2.3, specific wear rates are calculated based on four local measurements using WLI method. Therefore, depending on variation in volume loss from the four different locations, a standard deviation can be defined for specific wear rate measurement corresponding to each wear track. This deviation is a measure of uncertainty associated with specific wear rate determination due to local variations within the wear track. The standard deviation values (*Sm*) for all the radii are reported as error bars in Figure 3. It can be seen that a standard deviation as high as 4.18 mm^3^·N^−1^·m^−1^ can occur in specific wear rates corresponding to the same wear track, calculated based on four local measurements. It is also noticeable that no trend is evident in magnitude of *Sm* by changing angular velocity, which means the number of revolutions on the same wear track as well as angular velocity does not significantly influence uniformity of the wear track for the radii between 5 and 9 mm and angular velocity corresponding to 1333 rpm to 2400 rpm. This indicates that the effect on uniformity of wear track due to change in angular velocity can be considered negligible within this window.

#### 3.2.2. Variation in Specific Wear Rate

Figure 3 shows specific wear rate value for all the 15 test runs (3 each for the five track radii tested), and the error bars indicate standard deviation (*Sm*) of the four measurements on each wear track.

Barring S2R5, S1R7 and S3R8, at least two out of the three tests performed at each radius on the three samples were found to result in similar specific wear rate values. Regardless of the different wear track radii, a clear overlap in error bars can also be recognized. Therefore, the three conditions above (S2R5, S1R7 and S3R8) may be considered to be outliers. Thus, the ball-on-disk tests are observed to yield consistent specific wear rate results regardless of the angular velocity being varied over a wide range, as demonstrated by the present experiments. This is due to the fact that no change in wear mechanisms and wear regime happened in this window of tests conditions. More detailed discussion is provided in Section 3.5.

**Figure 4:**
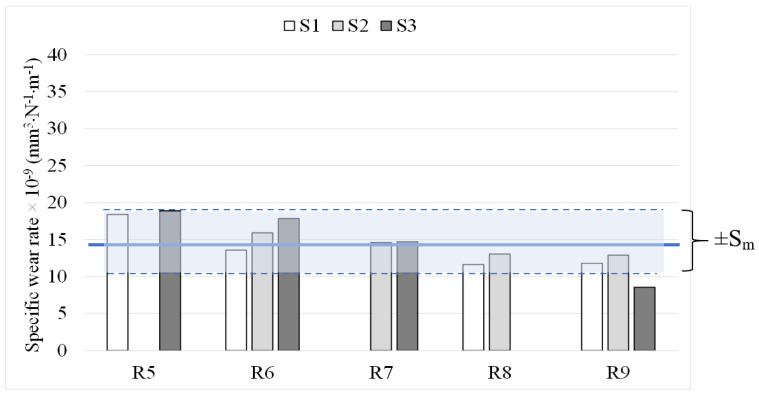
Average specific wear rate value of different wear track radii.

To further highlight the consistency of the specific wear rate results regardless of angular velocity, omitting the three above mentioned outlier values, the calculated average value of the specific wear rate and the maximum standard deviation that can occur from measurement are depicted in Figure 4.

It can be seen that all the average values of each radius fall in a range of around 11—18.6 mm^3^·N^−1^·m^−1^. This deviation in average specific wear rate is smaller than the maximum standard deviation from measurement (Max. *Sm* = ±4.18 mm^3^·N^−1^·m^−1^). Therefore, it can be concluded that maximum deviation from changing the wear track radius (and, hence, angular velocity) is smaller than the maximum standard deviation from the measurement method itself. Moreover, standard deviation of the specific wear rates of all the twelve measurements is 2.9 units, which is still much less than maximum *Sm*. Therefore, results from the tests performed on the same sample with various track radii (between 5 and 9 mm in the present study and corresponding to substantially varying angular velocity) can be consistent and deemed to be representative of wear behaviour of the coating.

#### 3.2.3. Variation in Coefficient of Friction and Wear Mechanism

**Figure 5:**
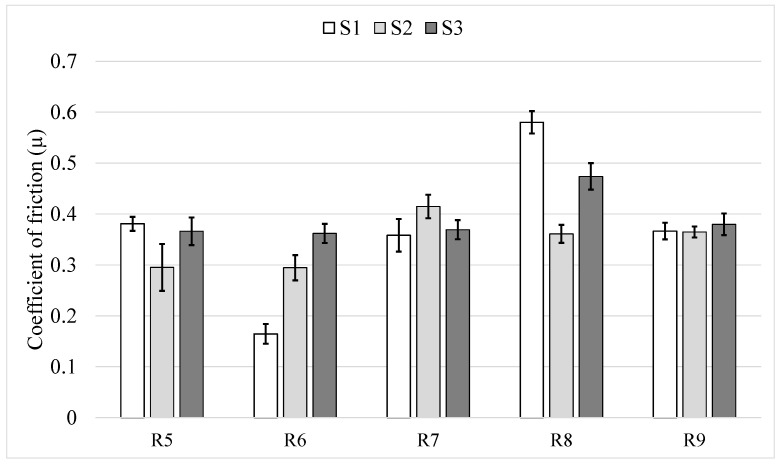
Average CoF value for all the fifteen tests.

The coefficient of friction (CoF) for all the samples reached steady state after around initial 5000 s of sliding. Figure 5 shows average steady state CoF values for all the 15 test repetitions performed on the 3 samples at different radii, along with the standard deviation (as error bars) showing fluctuation of CoF values. It can be seen that a majority of CoF values fall into a narrow range of 0.3 to 0.4. However, looking at S1R6 and S1R8, the average CoF value can deviate from 0.16 to 0.58 units without any considerable change in the specific wear rate (see Figure 3). Comparing specific wear rate figures with CoF values (Figure 3 and Figure 5), no direct correlation can be clearly made between specific wear rate and its corresponding friction coefficient value. Unlike two studies conducted by Wesmann et al. [29,30] attributing higher coefficient of friction to formation of surface oxides and tribofilm, no obvious correlation was identified between CoF values and surface morphology during post-wear analysis. In other words, some wear tracks were found to be similar, while the corresponding CoF values were different by a factor of 2 or 3. Hence, the CoF value in the WC–CoCr coating studied in this paper appears to vary without a clear trend within the aforementioned range. Similar large variation in CoF without any clear trend was found in a study by Wilkowski et al. [31].

**Figure 6:**
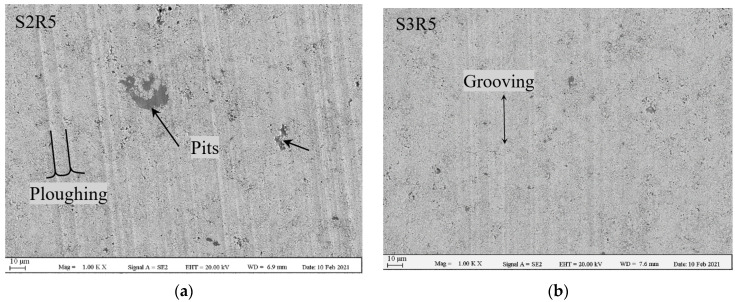

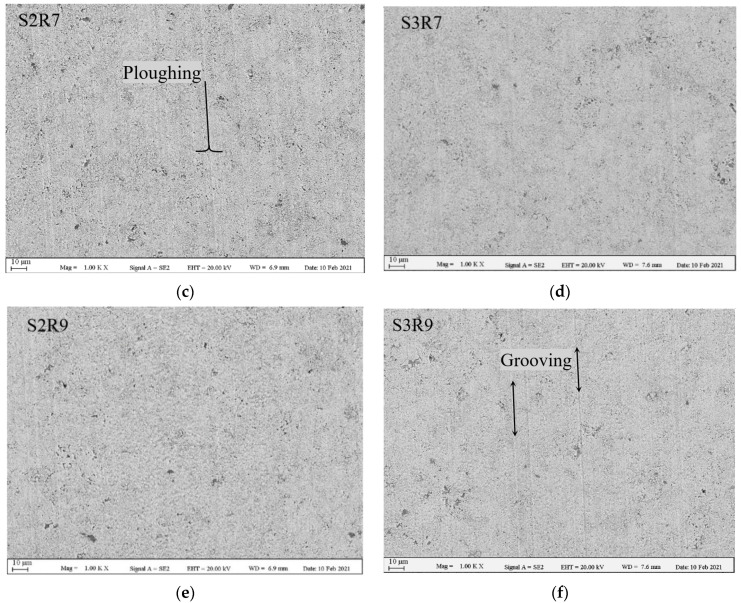
SEM images of wear tracks at different radii of 5, 7 and 9 mm. (**a**) S2R5; (**b**) S3R5; (**c**) S2R7; (**d**) S3R7; (**e**) S2R9 and (**f**) S3R9.

Although the specific wear rate values appear consistent regardless of angular velocity as discussed above, it is also important to ensure that this observation is not coincidental and there is no change in wear regime over the test parameter window under consideration. Therefore, in order to investigate the wear mechanisms responsible for material removal, wear tracks were studied under SEM. Figure 6 shows SEM images of wear scars of various radii of R5, R7 and R9 on the two samples, S2 and S3. Ploughing is the dominant wear mechanism regardless of the angular velocity of the test. In addition, some signs of shallow grooving can be detected (shown by double sided arrows). These two mechanisms are also reported in other studies to be the common wear mechanisms in case of WC-based coatings under sliding wear conditions [26,32]. SEM image on wear scar of the sample S2R5 shows that the number and depth of the ploughs are extremely higher than the rest of the test runs, which is the reason for its specific wear rate being too high (see Figure 3). Moreover, as shown by arrows, there are regions with significant material removals within this wear track, while no such large-scale removal (in form of pitting wear) was detected in other wear tracks. The reason for this unique behaviour was not clear. By comparing SEM images of the wear tracks with various radii (various angular velocities), it is clear that the quantity and depth of ploughs and grooves are differing randomly from a case to the other, without any direct correlation with the radius (angular velocity). For instance, number of ploughs on the sample S2R7 are clearly higher than the sample S2R9, but this trend is completely reversed in the case of sample 3 (compared S3R7 with S3R9). All things considered, it is clear that for none of the samples major wear mechanisms changed, and it is dominated by ploughing and minor grooving.

### 3.3. Influence of Load on Wear Behaviour

#### 3.3.1. Variation in Specific Wear Rate

**Figure 7:**
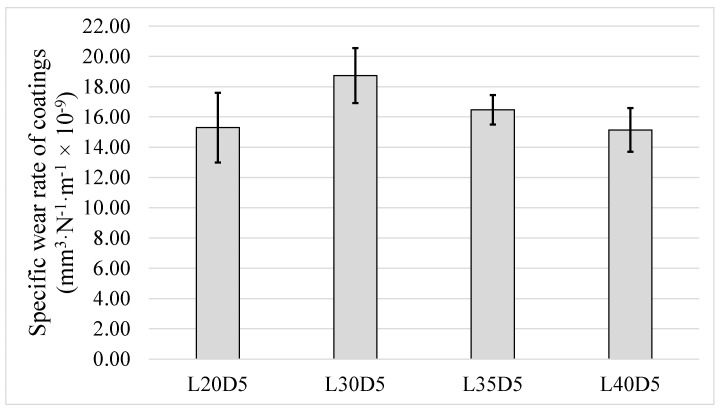
Specific wear rates from ball-on-disc tests conducted under different loads.

Wear behaviour of the HVAF-sprayed WC–CoCr coating was also evaluated at different loads applied during ball-on-disk testing. Specific wear rate values corresponding to various loads are reported in Figure 7. No major change in specific wear rate was detected, and it is also clear that no major variation in the range of error bars is noted. This means that increase in load over the range investigated (20 N to 40 N) has no influence on consistency of the sliding wear test results.

#### 3.3.2. Variation in Coefficient of Friction and Wear Mechanism

**Figure 8:**
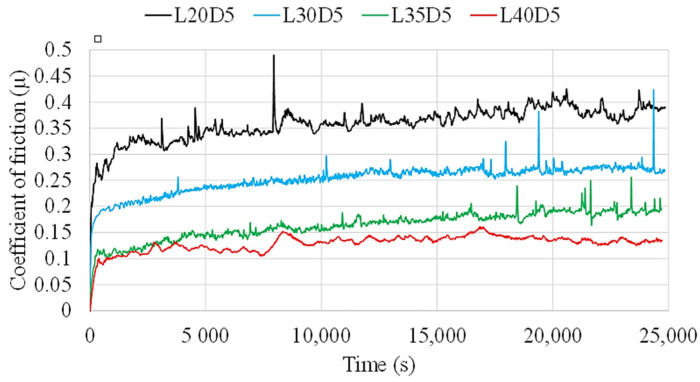
CoF evolution for the coating tested under different normal loads.

Figure 8 shows evolution of friction coefficient of the samples during the tests performed at radius of 7 mm. The height of the CoF plateau is observed to clearly decrease with increasing normal load. A gradual decrease in the average values can be seen when increasing the load from 20 N to 30 N, 35 N and 40 N. This indicates that, in spite of increase in normal load, friction load does not experience any considerable change. Although some studies can be found reporting a similar trend [33], this trend can also exhibit random behaviour depending on contact conditions [26] or may even be completely reversed [34] based on the number of third-body particles involved in the contact region. In general, when fewer third-body particles are involved in the test, i.e., when the test condition is close to two-body wear, with increase in load, the CoF value decreases.

**Figure 9:**
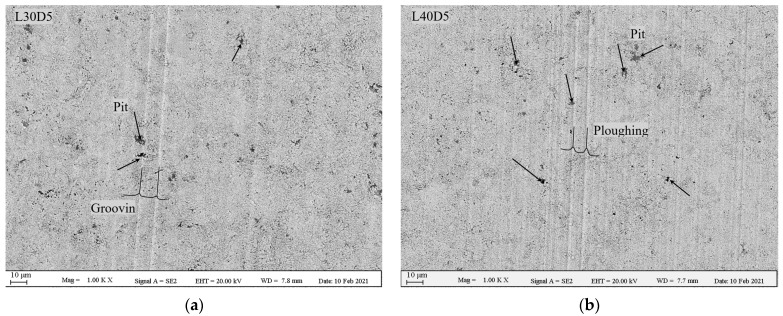
SEM images of wear tracks at different normal loads of 30 N and 40 N. (**a**) L30D5 and (**b**) L40D5.

To investigate any possible change in mechanism of wear as a result of increase in load, SEM analysis was performed on wear tracks. Figure 9 shows SEM images of wear scars of the L30D5 and L40D5 samples. The number of ploughs has clearly experienced a noticeable increase by increasing the load from 30 N to 40 N. Comparing Figure 6 and Figure 9, it is clear that, in spite of the increase in load from 20 N to 40 N, ploughing is still the dominant wear mechanism. This suggests that no significant changes occurred in the mechanism of material removal [1,26,35].

### 3.4. Influence of Sliding Distance on Wear Rate

**Figure 10:**
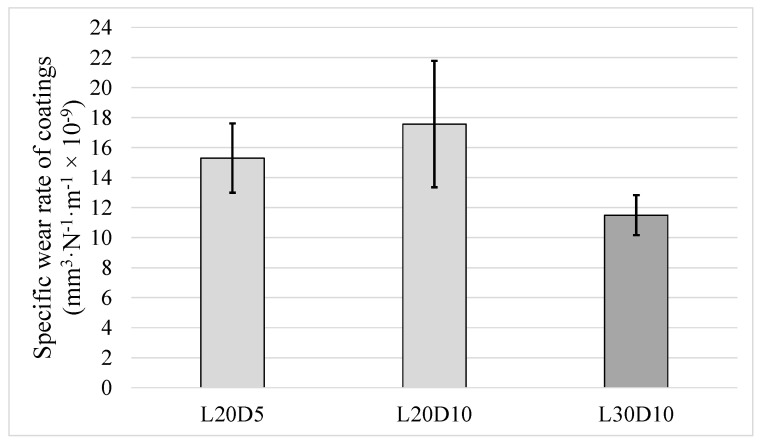
Specific wear rate values from ball-on-disc tests conducted for different sliding distance and load.

Running the test for longer time (longer sliding distance) is another way to investigate any changes in wear rate. Three tests were conducted for this matter: two with the same load of 20 N and different sliding distance of 5 km and 10 km (L20D5 and L20D10) and one with normal load of 30 N and distance of 10 km (L30D10). Figure 10 shows specific wear rate of the three samples. Increasing sliding distance of the test from 5 km to 10 km (under normal load of 20 N) resulted in neither a substantial difference in specific wear rate nor any improvement in repeatability of result (bigger error bar for L20D10 compared to L20D5). As presented later in Figure 12, all the three values are still within the window of maximum *Sm* and thus do not differ significantly.

**Figure 11:**
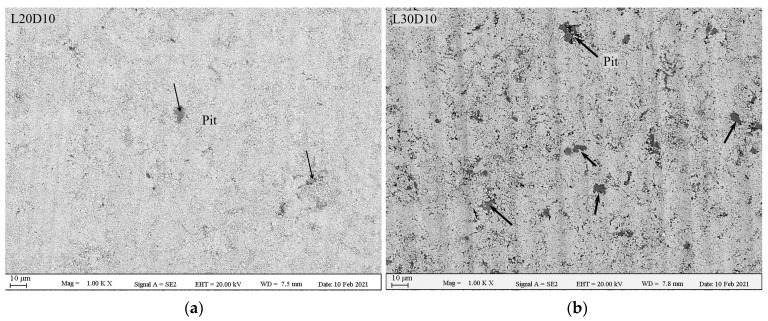
SEM images of wear tracks performed under 20 N (**a**) and 30 N load (**b**).

Figure 11a,b show SEM images on wear tracks of the two L20D10 and L30D10 samples. First, by comparing Figure 11a and SEM images in Figure 6, it is clear that no major changes are detectable by increasing the sliding distance from 5 km to 10 km under the same normal load of 20 N. However, a noticeable change is evident when comparing SEM images of Figure 11a,b, which is rooting from an increase in applied normal load from 20 N to 30 N when running for the same distance of 10 km. One clear change is that quantity and depth of the ploughs experienced an obvious increase, and the number and size of pits (dark regions) also considerably increased. The pits, which are filled up with wear products, indicate material removal [18]. Therefore, by a simultaneous increase in the normal load (from 20 N to 30 N) and sliding distance (from 5 km to 10 km), some signs of material removal in form of pits is added to ploughing as dominant wear mechanisms. However, as far as the pits are isolated and therefore possible to be filled up with wear products such as oxides of alumina, tungsten, cobalt and chromium, they are not taken into account in volume loss measured by WLI technique and, consequently, not in specific wear rate. This explains no major change in specific wear rate value reported in Figure 10 in spite of minor change in removal mechanisms and, therefore, no changes in wear regime. As shown in the study by Wang et al. [26], connection of these pits can cause a dramatic change in removal mechanism and result in the magnitude of specific wear rate.

### 3.5. Wear Regime

**Figure 12:**
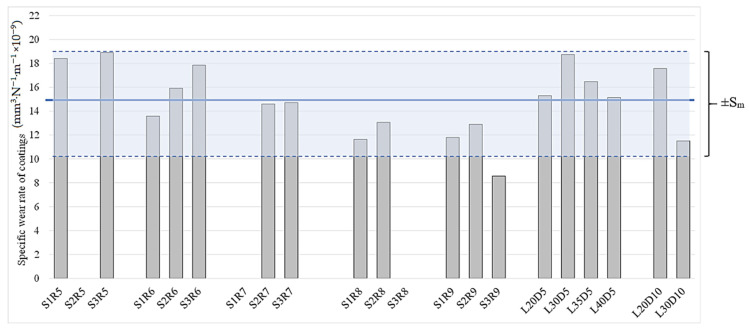
Specific wear rate from various ball-on-disc tests conducted under different conditions according to Table 3.

**Figure 13:**
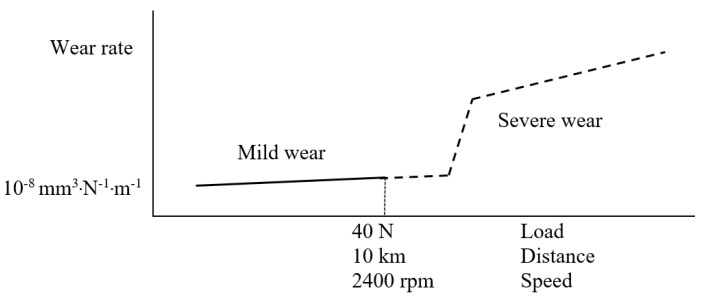
Schematic diagram of the mild and severe wear regimes adapted from [1].

When the specific wear rate is low and does not substantially change (i.e., remains of same order of magnitude) by changing test conditions, the wear regime can be termed as mild [1,26,35]. Moreover, in case of WC-based coatings, it is known that, after a certain increase in normal load, the specific wear rate experiences a substantial increase by orders of magnitude [26]. The specific wear rate values for various angular velocities, loads and sliding distances (from a large matrix of ball-on-disk tests conducted, as listed in Table 3) previously presented in Figure 4, Figure 7 and Figure 10 reveal that the average specific wear rate values vary from 11 to 18.6 mm^3^·N^−1^·m^−1^, which are small enough to be categorized as mild wear regime. All the specific wear rate values from ball-on-disc testing under different angular velocities, different loads and/or sliding distances along with the corresponding average value are depicted in Figure 12. It can be seen that deviation of all the specific wear rate values (with the only exception of S3R9) from average value is still within the window of maximum *Sm*. It should be mentioned that the conclusions in this paper are specific to sliding wear rate behaviour of HVAF-sprayed WC–CoCr coatings, and the results may differ for other coatings. Additionally, no significant difference in wear mechanisms was evident from post-wear analysis, confirming that no transition from mild to severe wear regime occurs over the investigated test parameter window. Therefore, as shown in Figure 13, by individually increasing either normal load up to 40 N, angular velocity up to 2400 rpm, or sliding distance up to 10 km from the ‘reference’ test parameters of 20 N, 2400 rpm and 5 km, no changes in wear regime occur during ball-on-disk testing of HVAF-sprayed WC–Co-Cr coatings.

A comprehensive investigation was conducted in order to understand sliding wear behaviour of HVAF-sprayed WC–CoCr coatings under a variety of different test conditions, and the following conclusions were drawn: No significant difference was observed in specific wear rate when changing angular velocity from 1333 rpm up to 2400 rpm in a way that the difference in smaller than precision of measurement method. Increase in load up to 40 N did not make any major change in either specific wear rate or deviation of values from different repetitions. Increase in sliding distance up to 10 km while the normal load was fixed at 20 N changed neither specific wear rate nor repeatability deviation. All these roots from no major change in dominant wear mechanisms or wear regime. Therefore, conducting ball-on-disc test on WC–CoCr coatings under test conditions within the range conducted by this study could be considered as a reliable testing routine.

## Figures and Tables

**Table 1:** Characteristics of WC–CoCr feedstock powder.

Composition (% Mass)		Particle Size Range (µm)	Carbide Size	Service Temp. (°C)
Co: 8.5–11.5 Cr: 3.0–5.0	C: 5.0–6.0 W: Bal.	5/30	fine	<500

**Table 2:** Grit-blasting and spraying parameters employed.

	Gun/Nozzle	Air (psi)	Fuel 1 (psi)	Fuel 2 (psi)	Carrier (L/min)	Feed (g/min)	SoD (mm)	Number of Strokes
**Grit-blasting**	M3/5L2	110	100	80	60	≈100	350	2
**WC–CoCr**	M3/5L2	118	105	115	50	200	300	16

**Table 3:** Test parameters employed during sliding wear tests.

Variable Parameter	Code	Radius (mm)	Load (N)	Distance (km)
**Angular velocity**	R5 (S1/S2/S3)	**5**	20	5
	R6 (S1/S2/S3)	**6**	20	5
	R7 (S1/S2/S3)	**7**	20	5
	R8 (S1/S2/S3)	**8**	20	5
	R9 (S1/S2/S3)	**9**	20	5
**Load**	L20D5	7 and 8	**20**	5
	L30D5	7 and 8	**30**	5
	L35D5	7 and 8	**35**	5
	L40D5	7 and 8	**40**	5
**Distance**	L20D10	7 and 8	20	**10**
**Load and distance**	L30D10	7 and 8	**30**	**10**

## Data Availability

Data is contained within the article.
